# Colonization of a Deglaciated Moraine: Contrasting Patterns of Carbon Uptake and Release from C3 and CAM Plants

**DOI:** 10.1371/journal.pone.0168741

**Published:** 2016-12-29

**Authors:** Elisa Varolo, Damiano Zanotelli, Leonardo Montagnani, Massimo Tagliavini, Stefan Zerbe

**Affiliations:** 1 Faculty of Science and Technology, Free University of Bozen/Bolzano, Bolzano, Italy; 2 Institute of Biology and Chemistry, University of Hildesheim, Hildesheim, Germany; 3 Forest Services, Autonomous Province of Bolzano, Bolzano, Italy; Pacific Northwest National Laboratory, UNITED STATES

## Abstract

**Introduction:**

Current glacier retreat makes vast mountain ranges available for vegetation establishment and growth. As a result, carbon (C) is accumulated in the soil, in a negative feedback to climate change. Little is known about the effective C budget of these new ecosystems and how the presence of different vegetation communities influences CO_2_ fluxes.

**Methods:**

On the Matsch glacier forefield (Alps, Italy) we measured over two growing seasons the Net Ecosystem Exchange (NEE) of a typical grassland, dominated by the C3 *Festuca halleri* All., and a community dominated by the CAM rosettes *Sempervivum montanum* L. Using transparent and opaque chambers, with air temperature as the driver, we partitioned NEE to calculate Ecosystem Respiration (R_eco_) and Gross Ecosystem Exchange (GEE). In addition, soil and vegetation samples were collected from the same sites to estimate the Net Ecosystem Carbon Balance (NECB).

**Results:**

The two communities showed contrasting GEE but similar R_eco_ patterns, and as a result they were significantly different in NEE during the period measured. The grassland acted as a C sink, with a total cumulated value of -46.4±35.5 g C m^-2^ NEE, while the plots dominated by the CAM rosettes acted as a source, with 31.9±22.4 g C m^-2^. In spite of the different NEE, soil analysis did not reveal significant differences in carbon accumulation of the two plant communities (1770±130 for *F*. *halleri* and 2080±230 g C m^-2^ for *S*. *montanum*), suggesting that processes often neglected, like lateral flows and winter respiration, can have a similar relevance as NEE in the determination of the Net Ecosystem Carbon Balance.

## Introduction

Worldwide, glaciers are melting owing to increases in global temperature [1]. The decreasing albedo [2] and the enhanced methane emissions from the melting permafrost [3] [4] represent relevant positive feedbacks to the current increase in global greenhouse gasses (GHG) concentrations and to the related radiative forcing. However, shortly after ice melt, vegetation establishes on the deglaciated area and a new ecosystem begins to develop on the glaciers forefield. The plant colonization of exposed terrain and the increasing growth in the higher latitude and altitude [5,6] possibly represent a negative feedback to the increase in global GHG concentrations.

Besides its relevance, to date, few studies have analysed the carbon (C) budget in glacier forefields [7,8]. The C budget of an ecosystem is described through the Net Ecosystem Exchange (NEE), also referred to as Net Ecosystem Production (NEE = -NEP), which represents the difference between C assimilation, Gross Ecosystem Exchange (GEE), and C release, Ecosystem Respiration (R_eco_)[9–11]. The NEE provides an index of the ability of an ecosystem to fix (if negative) or to lose (if positive) C due to photosynthesis and respiration, and is generally considered at short time scales. At longer time scales, non-gaseous C losses frequently become more important. In a long-term perspective, to define the C budget of an ecosystem we should also consider the C losses e.g. through leaching of organic (DOC) and inorganic C (DIC) [12], as well as non CO_2_ fluxes and lateral C fluxes (in and out) from bordering ecosystems [13]. The ultimate amount of C accumulated by any ecosystem in a given time period that also accounts for these terms is defined as the Net Ecosystem Carbon Balance (NECB)[14].

Studies on the C budget of ecosystems close to glacier forefields, such as alpine grasslands [15], or ecosystems with similar vegetation, such as arctic tundra [3], indicate that they can act either as a sink or a source of C [16]. In fact, in grassland ecosystems, climatic drivers have a strong influence on the assimilation and emission processes, thus leading to large inter-annual variability [17].

Moreover, by analysing the C budget of a glacier forefield we have to consider that we have to deal with an extremely patchy and dynamic ecosystem [18]. The development of vegetation on glacier forefields can take multiple directions as soil conditions and environmental conditions can be rather heterogeneous [19]. As a result, newly established vegetation is patchy distributed and may differ in character [20]. Rocky surfaces are mostly colonized by lichens, whilst mosses and C3 grasses prevail on sandy soils and rosette plants with CAM metabolism often colonize gravel soil [21]. It is still unknown to what extent the presence of different plant communities in the same ecosystem affects the final NECB: species interact in different ways with the other components of an ecosystem [22].

Several studies have compared C fluxes and final NECB from different vegetation communities, focusing on different characteristic of the community: species composition, plant traits, physiology of the main species etc. Glenn et al. [23], on studying the CO_2_ exchange in two different vegetation types in Canadian bogs, found that the site dominated by *Sphagnum*, in spite of its lower maximum assimilation capacity during summer, had a larger overall C sink than the site dominated by *Carex*. Bubier et al. and Glenn et al. [23–25] studied the CO_2_ exchange in peatlands and found contrasting effects of the water table on the different plant functional groups. Hirota et al. [26] analysed the NEE in a Tibetan grassland, and showed that plant biomass and species richness had a greater effect on NEE than species composition. In different experiments, Wohlfahrt et al. [27] Ward et al. [28,29], observed that species composition had a larger effect on the C budget than did canopy structure or experimental warming. Christensen et al. [30] studied the assimilation and emission processes in different tundra vegetation communities through the combined use of opaque and transparent chambers, and found that the differences in NEE rates were related to plant biological characteristics.

The study of C assimilation and release in plant communities differing in their photosynthetic pathways is particularly interesting as C3 species and CAM plants differ in their mechanism of fixing atmospheric C. C3 species assimilate CO_2_ during the day, when stomata are open, and directly fix C through the enzyme Rubisco during light conditions. CAM plants use Phosphoenolpyruvate carboxylase (PEPC) in addition to Rubisco for carbon fixation. This allows a more flexible use of stomatal openness to assimilate and release carbon dioxide and water vapour.

There is a distinction between full (obligate) CAM plants that firmly assimilate CO_2_ during the night through PEP and weak (facultative) CAM plants that can switch from CAM to C3 metabolism[31,32]. In obligate CAM plants, generally found in desert conditions, net CO_2_ uptake occurs predominantly during the night, when they first fix CO_2_ by PEPC and store it as malic acid. Then, during the day, when stomata are closed, the malic acid is broken down and C is fixed by the normal C3 pathway [33]. In facultative CAM plants, growing from the tropics to the alpine environment, a wide variety of combinations of daily patterns of assimilation and release of CO_2_ and H_2_O can be found [34,35]. As a result of the ontogenetic process and meteorological influences, facultative CAM plants display a very plastic response to the environment and can finely modulate the intensity of C assimilation through PEP during the night or Rubisco during the day in response to the climatic characteristic of the external ambient and the internal conditions of the plant [36]. As the CAM metabolism increases water use efficiency, CAM plants are found on alpine sites in higher altitudes only in specific micro-habitats characterized by particular water or heat stress, where C3 species are excluded[21].

In this study, we give a contribution in the analysis of the C budget of recently deglaciated area by investigating if the presence of different vegetation communities influences the C budget of the ecosystem. Therefore, we investigated the CO_2_ fluxes of two well-differentiated plant communities living on the same stage of a primary succession on the Little Ice Age (LIA) moraine at 2400 m a.s.l. on the southern slopes of the Alps. One was a typical C3 grassland of glacier forefields, *Festucetum halleri* association after Braun-Blanquet and Jenny (1926). The second was a community mainly composed of *Sempervivum montanum* L., a succulent species exhibiting a weak CAM metabolism[21,37], commonly found on rocky and dry soils. NEE data collected were used to answer the following questions:

1. are we able to quantify assimilation (GEE) and release (R_eco_) processes in the two vegetation communities?
2. In the short time period, which is our measured growing season, do C3 and CAM plant communities have a different cumulated NEE?
3. in the long term period, time since glacier retreat, what is the NECB of the glacier forefield ecosystem?
4. to determine its C budget, is it important to consider the presence of different vegetation communities on the deglaciated area?

## Material and Methods

### Study area

**Fig 1:**
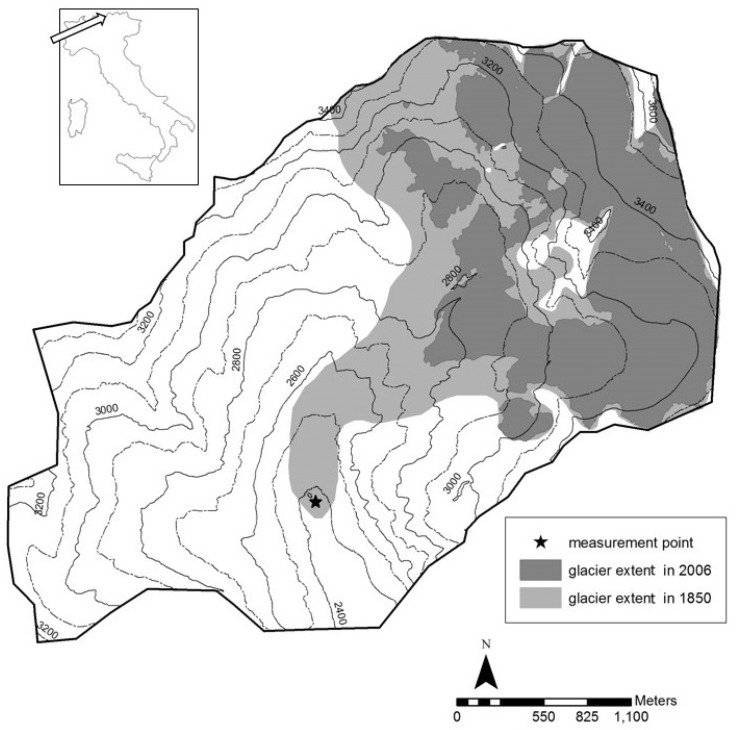
Study area in the upper Matsch valley (Northern Italy). The glacier extent in 2006 and 1850 is shown. The star indicates the site of measurements.

The study was conducted in the upper catchment area of the Matsch valley, which has a drainage area of approximately 11 km^2^. The Matsch valley is enclosed within the upper Vinschgau valley in the Province of Bozen located in the Italian Alps (Fig 1). The Forest Service of the Autonomous Province of Bolzano granted the permission to reach the site and to take the samples. This study did not involve any endangered or protected species. From a geological point of view, the Matsch valley belongs to the Ötztal-Stubai complex and consists mainly of Orthogneis [38]. The bottom of the valley has a relatively dry climate with an annual average rainfall of 550 mm (1970–2000). Rainfall increases with altitude and reaches even 1000 mm in the upper catchment part. The annual average temperature at 1570 m above sea level (a.s.l.) is 6.6°C [39].

The Matsch glacier is a part of the Palla Bianca-Weisskugel Glacier complex and is located between 2,700 and 3,700 m a.s.l. In 1850, at the end of the Little Ice Age (LIA), the maximal extent of the Matsch Glacier was 4.88 km^2^, and in 2006 it had decreased to 2.78 km^2^ [40]. The study site lies at 2,400 m a.s.l., and has been free of ice for about 160 years. The primary succession on the Matsch glacier forefield is similar to that of nearby localities as described by Raffl and Erschbamer [20]. The colonization process can be observed at a few meters from the glacier tongue, with mosses and single individuals of *Leuchantemopsis alpina* L. and *Arenaria biflora* L. On the moraine ridges of 1940, a pioneer grassland community, dominated by *Poa laxa* Haenke, *Gnaphalium supinum* L., *Cerastium cerastoides* (L.) Britton and *Arenaria biflora* L. is present and covers about 35% of the surface area. On the LIA moraine, the vegetation covers about 70% of the soil and presents different vegetation communities. These patchy vegetation groups, which have a lower rate of habitat disturbance, are represented for example by *Festucetum halleri* after Braun-Blanquet & Jenny (1926), the community composed mainly of the succulent species *Sempervivum montanum* L., and the *Cetrario-Loiseleurenion* alliance after Braun-Blanquet et al., 1939. Beyond the moraine ridges, the vegetation of the upper Match valley is represented, on the valley bottom, by the grass meadow *Nardion strictae* (Braun-Blanquet & Jenny, 1926), and along the sides of the valley by the shrub community *Rhododendro-Vaccinion* (Braun-Blanquet & Jenny, 1926).

### Meteorological data

Air temperature and relative humidity were measured at 0.1 and 2 m above ground level (RHT Plus, Skye Instruments, Llandrindod Wells, UK) along with global radiation (measured at 0.1 m with a model CM6b pyranometer, Kipp & Zonen, Delft, Holland), and wind speed (measured at 2 m above the ground with an A100r anemometer Vector Instruments, UK). Soil temperature and soil relative humidity were measured at 5 cm below the soil surface with a PT100 and 10HS, Decagon, Pullman, WA, USA, respectively, and collected using the Li 8100 system (Li-Cor Biosciences, Lincoln, NE, USA, Li-Cor hereafter). Measurements were taken every 10 s and collected at 10 min intervals by a DL2 datalogger (Delta Devices, Cambridge, UK). All data were then averaged at 30-minute time steps.

### NEE measurements

In 2012 and 2013, NEE was measured on two typical plant communities in light and dark conditions using the chamber technique. The two communities were chosen because of the different photosynthetic pathways of the two dominant species, which were *Festuca halleri* All. (hereafter, termed *Festuca* plots) and *Sempervivum montanum* L. (hereafter, termed *Sempervivum* plots). The two communities were closely located in many small independent units inside an area of approximatively 30 m of diameter. On the chaotic disposition of morenic sediments, the two communities occupied slightly different microhabitats: in small dips with sand accumulation we found *Festuca* plots, on stonier and hilly places *Sempervivum* plots. The measurement system consisted of an infrared gas analyser (LI-8100, Li-Cor) connected to a multiplexer (LI-8150, Li-Cor) which allowed the combined use of eight automated closed-dynamic chambers: four transparent (LI-8100-104C, Li-Cor) and four opaque ones (LI-8100-104, Li-Cor). The transparent chambers were used to measure the gas exchange of the selected ecosystems during ambient light conditions, while the opaque ones were used to measure the gas exchange under dark conditions. A rotational technique (see below) was used to measure gas exchange under light and dark conditions on the same collars, which enabled partitioning of photosynthetic and respiratory fluxes and provided an accurate C-balance measurement [41].

In 2012, measurements were carried out during the peak vegetative growing season for 18 days (28 July—14 August 2012) and aimed to characterize the short-term variability of CO_2_ fluxes of two representative vegetation communities with different photosynthetic pathways. Five *Festuca* (F1, F2, F3, F4, F5) and five *Sempervivum* (S1, S2, S3, S4, S5) plots were selected for light and dark NEE measurements. As we could use only eight chambers (4 transparent and 4 opaque) simultaneously, we resorted to a rotational measurement scheme. In every measurement period, which lasted three days, we measured light and dark NEE from two replicates in each vegetation type. After three days, the collars were removed and two new replicates were measured. This scheme was followed throughout the 18-day measurement campaign (S1 Fig). To minimize the disturbance effect due to installation [42], data collection in both years started two weeks after the ten iron collars (20 cm diameter, 8 cm depth) were placed in the soil.

In 2013, measurements were carried out to assess the seasonal variability of the fluxes and the C balance during the whole vegetation season. Measurements were taken from 6^th^ June, just after snowmelt, until 17^th^ September, after the first relevant snowfall, totalling 103 days and covering most of the C-uptake period. We analysed the same 10 plots as in 2012, but with a different set-up: four chambers were kept on the same collar for the whole period (nearly four months), while the other four were rotated among the plots. Specifically, the long-term (LT) chambers measured one *Festuca* plot with an opaque chamber (FoLT); one *Festuca* plot with a transparent chamber (FtLT); one *Sempervivum* plot with an opaque chamber (SoLT) and one *Sempervivum* plot with a transparent chamber (StLT). The position of the other four chambers (two opaque and two transparent, connected to the same gas analyser and labelled as “short-term”—ST) was changed every week by rotating one transparent and one opaque chamber per vegetation type among the remaining plots (S2 Fig).

Measurement cycles were repeated at half-hour time steps, consistently with meteorological observations. They were composed of eight single chamber measurements taken in sequence. Each chamber measurement lasted 120 s + 30 s purge time, for a total of 20 minutes for each measurement cycle. We synchronized meteorological and flux measurements at 30 min time steps, and we assumed that half-hour averages of the meteorological conditions were representative of the two-minute period in which any single chamber measurement was taken. The exact chamber volume involved in flux calculation was computed every time the chambers were moved onto new collars by measuring the distance from the ground surface to the upper chamber basement at three regularly spaced points around the collar. For both opaque and transparent chambers, after the chamber closed on the collar, 20 s were considered as a mixing period, while the change in CO_2_ dry molar density during the following 40 s were used for the calculation of NEE (NEE = δ[CO_2_]/δt) with linear regression. The reduced time for flux computation was selected to minimize the effect of environmental perturbation inside the chamber. Although there is some evidence in literature concerning possible limitations of the linear regression [43], this model was preferred with respect to exponential fitting because the latter, when applied in the 40 s selected for flux computation, produced a significant amount of outliers, especially with clear chambers during daylight (S3 Fig).

### Carbon content analysis

After the flux measurements were concluded in 2013, total above-ground biomass was collected from each collar. This was divided into green biomass and necromass. A known volume of soil at an average depth of 10 cm was also taken from each collar. The exact volume after excavation was measured following Page-Dumroese et al. [44]. We put plastic balls into the hole until the balls were level with the top of the hole, then we weighed the balls required to fill the hole. The volume collected varied between 1000 and 1500 mL. Coarse roots (> 2 mm) were separated from the soil. Samples of above-ground biomass and roots were oven-dried at 60°C until the sample reached a stable weight. In addition, ten samples of the top 10 cm of mineral soil were collected a few meters away from the glacier tongue to determine the C content in the soil at the beginning of the colonization process (t_0_). Soil was oven-dried at 105°C until the sample reached a stable weight and then weighed and acidified with hydrochloric acid to eliminate the carbonate present [45]. The biomass and soil samples were analysed for total C and nitrogen content and for the C isotopic ratio (δ^13^C) with a FlashEA™ 1112 Elemental Analyzer (Thermo Fisher Scientific, Germany). The δ^13^C analysis was used to distinguish between photosynthetic pathways. During photosynthesis, the different enzymes involved in C assimilation discriminate against ^13^C, thus resulting in a different ratio of ^13^C/^12^C. Therefore, δ^13^C in C3 plants are known to range between -25 and -29‰, while C4 plants have a δ^13^C between -12 and -16 ‰. CAM plants have δ^13^C between -10 to -20‰ [46].

NECB is the organic carbon accumulation rate, described as the change in the organic C pool per unit time, and is usually expressed as mass of C per unit area for a specific time interval [47]. As our ecosystem appeared after glacier retreat at the end of the Little Ice Age, approximately 160 years ago, the NECB represents the organic C accumulated by the ecosystem over 160 years. To calculate NECB, the sum of C content found in the soil, above- and below-ground biomass, was measured from each collar and was compared with the C content in the soil at the beginning of the colonization process (t_0_).

### Data analysis

To analyse C flux data in *Sempervivum* plots and their relation to the environmental variables, we considered the four phases of CAM plants as described by Osmond [48]: phase 1 at night, carboxylation of atmospheric CO_2_ occurs through phosphoenolpyruvate (PEP) carboxylase and storage of C through the production of malic acid; phase 2 in the morning, carboxylation of atmospheric CO_2_ occurs through both PEP and ribulose bisphosphate carboxylase oxygenase (Rubisco); phase 3 during the central hours of the day, the decarboxylation of malic acid and the consequent re-fixation of C through Rubisco carboxylase occur; phase 4 during the afternoon, fixation of atmospheric CO_2_ occurs through Rubisco carboxylase. Osmond proposed the division of the daily pattern of CO_2_ fixation in four phases to help understand the CAM photosynthesis in full CAM plants. In facultative CAM plants, the activation of PEP carboxylase or Rubisco responds to a complex of internal and external factors. Therefore, the four phases could appear slightly modified: the phases could have a different duration, a time shift during the day, or some phases could be missing [34].

Photosynthesis in C3 plants, as *Festuca halleri*, clearly do not follow this four phases. C3 species have only two phases: during light condition, when stomata are open, C3 plants directly fix C through the enzyme Rubisco; in dark condition, they close the stomata to preserve water, and C fixation does not occur. However, in this study, we decided to divide the daily pattern of *Festuca* plots in the same time ranges as the four phases described for *Sempervivum* plots, in order to simplify the comparison between the CO_2_ fixation patterns in the two vegetation communities.

The data obtained in 2012 with both transparent and opaque chambers were used to present the daily courses of NEE in *Festuca* and *Sempervivum* plots. To characterize the plots, for each sampled collar we calculated the average, SD, minimum (Min) and maximum (Max) values of fluxes with both transparent and opaque chambers. Values were obtained on the half-hour flux data (μmol CO_2_ m^-2^s^-1^). The daily cumulated NEE (g C m^-2^d^-1^) was obtained as the sum of the grams of C exchanged every 30 minutes averaged for the three days of measurement of each plot. Day-to-day variability in the fluxes was reported as the average of range (max-min value) of every half-hour.

To verify the apparent response to light of the two vegetation communities, we applied a logistic sigmoid model [49] to NEE data measured with the transparent chambers in 2012:

$$NEE=2\cdot F_{\infty }\left(0.5-\frac{1}{1+\exp\left(\frac{-2\cdot \alpha \cdot PPFD}{F_{\infty }}\right)}\right)+R_{d}$$ (1)

where PPFD (photosynthetic photon flux density) is the driving variable, while *α* (initial quantum yield), *F*_∞_ (maximum NEE at full light) and R_*d*_ (daytime ecosystem respiration) are the three model parameters.

Due to the night CO_2_ uptake performed by CAM species, we could not approximate R_eco_ of the two vegetation communities using night values of NEE measured with the transparent chambers as customary methodology [50]. To model R_eco_, it is convenient to find a period when CO_2_ uptake does not occur. In CAM plants, and also in weak CAMs, this takes place only during the central period of the day under dark conditions. By analysing CO_2_ exchange data of the opaque chambers collected in both 2012 and 2013, we recognized that for both vegetation types, phase 3 of the day corresponded to maximum CO_2_ release and no CO_2_ assimilation over the entire measured season. Therefore, we considered NEE measured with opaque chambers during phase 3 being equal to R_eco_, and the same approach was applied for consistency both to CAM and to C3 plants.

An Arrhenius type model [51] was used to quantitatively assess the R_eco_ response function to air temperature of each measured plot by applying the following equation:

$$R_{eco}=R_{ref}\cdot e^{\left[E_{0}\cdot (0.0178507-\frac{1}{T+46.02})\right]}$$ (2)

where *R*_*ref*_ is the respiration flux at the constant reference temperature T_0_ = 10°C, *E*_*0*_ is an empirical parameter that indicates the temperature sensitivity of R_eco_ and T is the air temperature in °C. A two-sample Wilcoxon test was applied to check for statistically significant differences between the two plant communities with respect to the two parameters *R*_*ref*_ and E_0_.

To have a quantitative estimation of the differences in R_eco_ simulation using data of phase 3 of the day instead of using night data for C3 species, the entire 2013 dataset was firstly divided into four periods based on the reference month (from June to September). On those subsamples the model of Eq 2 was run using both nocturnal (phase 1) and diurnal (phase 3) data. Considering the four available replicates, there were no statistical differences in either *E*_*0*_ or *R*_*ref*_ (p-value two-sample Wilcoxon test > 0.05) and the difference in R_eco_ simulation ranged from a minimum of 0.16 g C d^-1^ in September to a maximum of 0.61 g C d^-1^ in July.

We analysed the data using a non-parametric statistical test because of the intrinsic uncertainty of normality tests (e.g. two sample t-test) when applied to a limited sample size (n = 4 per plant community type). Modelling efficiency (MEF) and residual standard error (RSE) were used to evaluate the accuracy of the curve fitting. MEF was calculated as follows:

$$MEF=1-\frac{\sum_{i=1}^{N}{\left(OBS_{i}-SIM_{i}\right)}^{2}}{\sum_{i=1}^{N}{\left(OBS_{i}-\bar{OBS}\right)}^{2}}$$ (3)

where *OBS*_*i*_ and *SIM*_*i*_ are the observed and simulated values respectively [52]. RSE is the square root of the error variance from the ANOVA table and is commonly given among the outputs when fitting a non-linear regression curve with the statistical software R (version 3.1.3, R Foundation for Statistical Computing, Vienna, AT), with which all statistical analyses were conducted.

Once the time series of R_eco_ values were established, the GEE was calculated according to the following definition equation:

$$GEE=NEE-R_{eco}$$ (4)

where NEE represents the net ecosystem exchange measured with transparent chambers. We assumed 0% of light-inhibition of autotrophic respiration [53]. For each flux component (GEE, NEE, R_eco_) we used the same micrometeorological convention, where negative values indicate net C uptake, and positive values indicate net C release from the ecosystem.

## Results

### Photosynthetic pathways and daily patterns of NEE

We investigated the δ^13^C in the biomass and soil of each sampled plot of the two vegetation communities (Table 1). The isotopic ratio in the above-ground biomass was significantly different in the two main species (Table 1, P = <0.001), thus highlighting the different photosynthetic pathways. The difference between the δ^13^C in the soil was P = 0.02 (Table 1).

**Table 1:** Comparison of isotopic ratio of carbon in the vegetation communities.

	*Festuca* plot	*Sempervivum* plot	*P value*
δ^13^ C soil	-26.18 ± 0.30	-25.74 ± 0.58	0.020
δ^13^ C biomass	-26.90 ± 0.47	-21.18 ± 0.41	< 0.001

Values are given in parts per thousand as averages of five samples ± standard error. P value of the ANOVA test with 12 (δ^13^C in soil) and 8 (δ^13^C in biomass) degrees of freedom.

**Fig 2:**
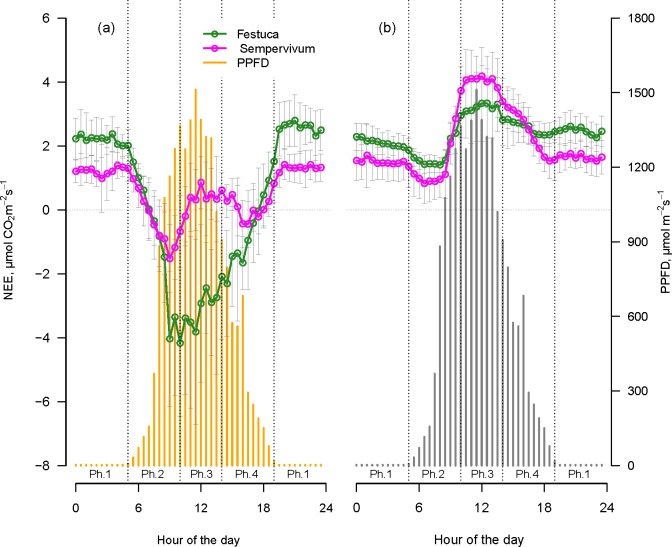
Average daily pattern (mean of three days of measurements ± SD) of net ecosystem exchange (NEE). Fluxes were measured in 2012 with (a) the transparent chambers and (b) the opaque chambers. Vertical bars indicate the average diurnal pattern of the photosynthetic active photon flux density (PPFD). Dotted lines split the day into the four CAM phases modified from Osmond [48] as follows:
Phase 1: the night; the period characterized by PPFD < 2 μmol m^-2^s^-1^.Phase 2: the morning; PPFD > 2 μmol m^-2^s^-1^ until phase 3 starts.Phase 3: the noon; the four central hours of the day, two hours before and two after the local midday.Phase 4: the afternoon; starting after phase 3 until PPFD < 2 μmol m^-2^s^-1^.

Table 2 shows the flux parameters, measured with both transparent and opaque chambers in 2012, characteristic for each plot. The daily average NEE measured with the transparent chambers in 2012 was 0.05 ± 0.32 g C m^2^ d^-1^ for the five *Festuca* plots, and 0.57 ± 0.23 g C m^2^ d^-1^ for the five *Sempervivum* plots. The average daily patterns of the measurements performed with the transparent chambers in 2012 were distinct for the two plant communities (Fig 2A). The *Festuca* plots presented the typical day and night photosynthesis cycle in C3 plants, where the net assimilation of CO_2_ occurs under light conditions with an assimilatory peak in the central hours of the day and CO_2_ emission during the dark period. The daily NEE trend of *Sempervivum* (Fig 2A) clearly followed the four phases as described by Osmond [48] for CAM plants. The measurements in this study showed that during phase 1, under dark conditions, *Sempervivum* plots emitted CO_2_, and only few records showed negative NEE values around 3 AM. During phase 2, a peak of negative NEE was recorded, thus proving assimilation, and the CO_2_ balance reached values around zero a few hours before midday. In the central part of the day, the *Sempervivum* plots emitted CO_2_ (phase 3) and reverted to CO_2_ assimilation in the final part of the light period (phase 4).

**Table 2:** Characterization of NEE fluxes measured for each plot with dark and clear chambers in 2012.

		NEE transparent chamber					NEE opaque chamber				
	Plot	Average	SD	Min	Max	Cumulated	Average	SD	Min	Max	Cumulated
		(μmol CO_2_ m^-2^s^-1^)				(g C m^-2^day^-1^)	(μmol CO_2_ m^-2^s^-1^)				(g C m^-2^day^-1^)
*Festuca* plots	F1	-0.19	3.17	-8.2	4.03	-0.132 (0.028)	2.84	0.99	1.26	5.56	3.215 (0.006)
	F2	0.67	3.8	-8.8	4.36	-0.369 (0.015)	2.56	0.51	1.28	3.78	2.644 (0.006)
	F3	0.02	1.88	-3.4	2.49	0.044 (0.011)	2.29	0.73	1.07	4.28	2.530 (0.010)
	F4	0.61	1.71	-3.4	3.03	0.390 (0.020)	2.21	0.63	1.22	4.58	2.260 (0.012)
	F5	0.22	1.82	-3.9	2.59	0.294 (0.016)	1.94	0.5	0.94	3.33	2.000 (0.008)
*Sempervivum* plots	S1	0.67	0.69	-2	1.99	0.737 (0.007)	1.93	1.13	0.17	5.18	1.977 (0.010)
	S2	0.14	1.32	-3.3	1.9	0.160 (0.012)	1.56	1.19	0.05	4.51	1.873 (0.007)
	S3	0.64	0.96	-2.5	2.14	0.661 (0.010)	1.92	1.24	0.6	5.2	2.360 (0.009)
	S4	0.62	1.23	-2.9	2.5	0.648 (0.014)	2.44	1.08	1.07	5.88	2.510 (0.014)
	S5	0.66	0.75	-1.8	2.07	0.687 (0.010)	2.41	1.02	0.77	5.29	2.454 (0.020)

The average cumulated value of daily NEE measured with opaque chambers in 2012 was 2.53 ± 0.46 g C m^-2^d^-1^ for *Festuca* plots and 2.23 ± 0.29 g C m^-2^d^-1^ for *Sempervivum* plots. Measurements conducted with the opaque chambers showed that *Festuca* displayed a peak of respiration during phase 3 of the day (Fig 2B). Similarly, *Sempervivum* also showed a peak of respiration in the same period of the day (Fig 2B), reaching higher emission values in comparison to *Festuca* plots. During the night, *Sempervivum* showed lower emissions than did *Festuca* plots.

Summarizing, in phase 2 of the day, *Festuca* plots reached the maximum assimilation value measured with the transparent chambers and in phase 3 the maximum CO_2_ emission measured with the opaque chambers. In *Sempervivum* plots, we observed two periods of high assimilation during the day with transparent chambers: phases 2 and 4. Maximum respiration occurred, as for *Festuca*, in phase 3.

Average, standard deviation (SD), minimum (Min) and maximum (Max) values are obtained on the half hour flux data (μmol CO2 m^-2^s^-1^). The daily cumulated value was obtained as the sum of the grams of C exchanged every 30 minutes averaged for the three days of measurements of each plot. Positive values of NEE indicate net C loss by the ecosystem. The day-to-day variability in the fluxes, reported in brackets, is represented with the average of range (max-min value) of every half-hour.

### NEE and solar radiation

**Fig 3:**
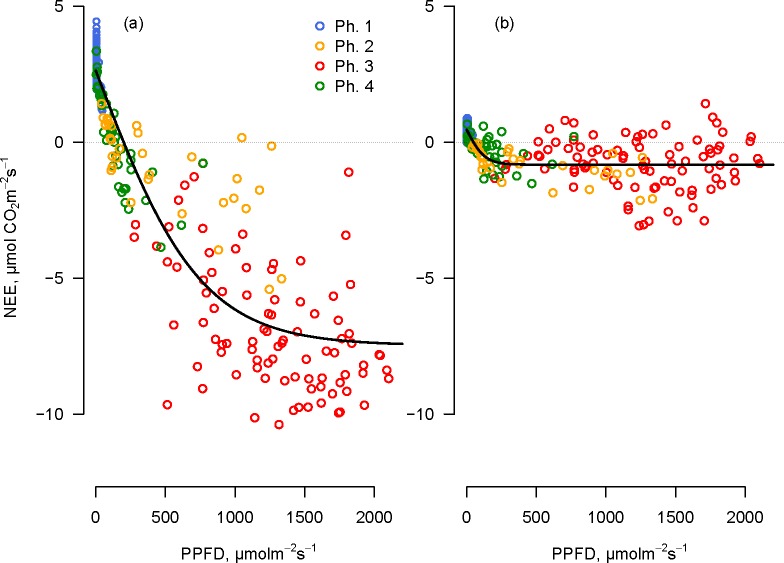
**NEE of (a) *Festuca* and (b) *Sempervivum* measured with clear chambers as a function of photosynthetic active photon flux density (PPFD).** Data reported correspond to a week of measurements in 2012 (7 August 2012–14 August 2012). The solid line indicates the apparent response to light obtained using Eq (1). Each color of the dots indicates one of the four “Osmond phases” in which the flux was measured, according to Fig 2 the four phases are presented for *Festuca* plots only with the purpose of simplifying the comparison between the two vegetation communities in the same time ranges.

Using the 2012 data, we analysed the apparent light sensitivity of NEE fluxes collected in transparent chambers. The most evident difference of NEE response to light between *Festuca* and *Sempervivum* plots was observed in the central part of the daylight period corresponding to Osmond’s phase 3. Higher net CO_2_ uptake was observed in *Festuca* plots than in *Sempervivum* plots (Fig 3). By modelling the photosynthetic light response according to Eq (1), we found that the three parameters α, *F*_∞_ and *R*_*d*_ were 0.013 ± 0.001, -10.100 ± 0.297 and 2.620 ± 0.130 for *Festuca* plots, while the parameters of the apparent response to light were 0.010 ± 0.002, 1.280 ± 0.077 and 0.450 ± 0.055 for *Sempervivum* plots. It is to be noted that owing to the existing decoupling between incident radiation and photosynthesis in CAM plants, observed parameters in the apparent response to light do not allow the reconstruction of the daily pattern of observed NEE in *Sempervivum* plots, but only in *Festuca* plots, where radiation and photosynthesis are tightly coupled in time.

### Reco and GEE modelling

**Fig 4:**
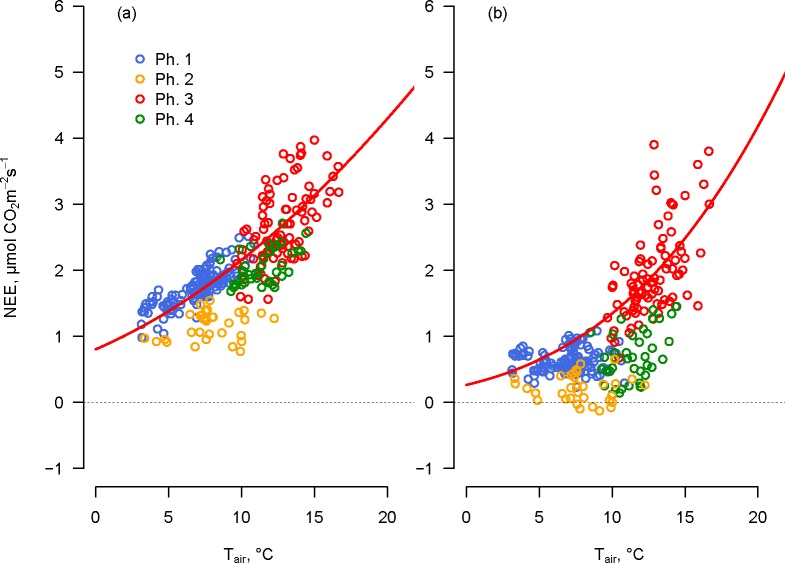
**Net ecosystem exchange (NEE) of (a) *Festuca* and (b) *Sempervivum* measured with opaque chambers as a function of air temperature.** Data reported correspond to a week of measurements in 2012 (7 August 2012–14 August 2012). Each colour of the dots indicates one of the four “Osmond phases” in which the flux was measured according to Fig 2. The four phases are presented for Festuca plots only with the purpose of simplifying the comparison between the two plants in the same time ranges. The solid red line represents the modelled NEE response to temperature obtained by applying Eq (2) only on data of phase 3, when we assumed that no CO_2_ uptake was occurring in the *Sempervivum* plots.

We calculated the relationship between NEE measured in 2012 and 2013 with the opaque chambers and air temperature for the two vegetation communities. During the day, the vegetation was acclimated to light until the closure of the chambers but as the chambers closed and for the entire duration of the measurement, vegetation was kept in dark condition. Measurements revealed that both vegetation types responded to temperature, but differed during the four Osmond phases in the *Sempervivum* plots (Fig 4). Plants in the C3 Festuca plots responded to temperature changes during all daily phases, while the CAM *Sempervivum* plots correlated with this driver only during the Osmond de-acidification phase 3. During the other phases, the relation between NEE measured with the opaque chambers and air temperature was weak, and in some occurrences during phase 2, acidification, the CO_2_ assimilation prevailed over the emission. Therefore, we decided to consider the NEE data measured with the opaque chambers during phase 3 only as R_eco_ for both communities. The relationship between R_eco_ and air temperature was calculated by applying Eq (2) to all the data of phase 3.

Results of respiration flux at the constant reference temperature T_0_ = 10°C (R_*ref*_), and temperature sensitivity (E_0_) for each replicate of *Festuca* and *Sempervivum* plots measured in 2013 are presented in Table 3. The values of MEF and RSE of every fitted curve indicate an overall good fitting of the selected model (Table 3). The average reference respiration at 10°C resulted in slightly higher values for *Sempervivum* than for *Festuca*. The average *R*_*ref*_ was 2.17 in *Sempervivum* and 1.83 μmol CO_2_ m^-2^ s^-1^ in *Festuca* plots, while temperature sensitivity was more pronounced for *Festuca* than for *Sempervivum* (the average of E_0_ was 206.53 and 179.80, respectively). However, the result of the two-sample Wilcoxon test showed no significant differences between the parameters, thus indicating that the two vegetation types were characterized by similar respiration patterns. The average *R*_*ref*_ and *E*_*0*_ parameters calculated for each vegetation type, combined with the half-hourly temperature dataset, were used to develop a vegetation-specific half-hourly R_eco_ estimate for the entire period measured.

**Table 3:** Respiration flux at the constant reference temperature *T*_*0*_ = 10°C (*R*_*ref*_) and the temperature sensitivity (*E*_*0*_)

	Plot	Parameters	Estimate	St. error	*P-value*	MEF	RSE μmol CO_2_ m^-2^ s^-1^
*Festuca* plots	Fo_ST_1	*R*_*ref*_	1.95	0.04	< 0.001	0.38	0.6
		*E*_*0*_	156.64	12.89	< 0.001		
	Fo_ST_2	*R*_*ref*_	1.84	0.08	< 0.001	0.72	0.71	
		*E*_*0*_	352.7	24	< 0.001			
	Fo_ST_3	*R*_*ref*_	1.6	0.03	< 0.001	0.52	0.37
		*E*_*0*_	182.32	14.95	< 0.001		
	Fo_LT_	*R*_*ref*_	1.92	0.04	< 0.001	0.22	0.79
		*E*_*0*_	134.45	11.89	< 0.001		
		*R*_*ref average*_	1.83	0.08			
		*E*_*0 average*_	206.53	49.7			
*Sempervivum* plots	So_ST_1	*R*_*ref*_	1.78	0.05	< 0.001	0.05	0.71
		*E*_*0*_	64.05	17.59	< 0.001		
	So_ST_2	*R*_*ref*_	1.86	0.11	< 0.001	0.47	0.97
		*E*_*0*_	282.26	32.25	< 0.001		
	So_ST_3	R_ref_	2.59	0.07	< 0.001	0.38	0.91
		*E*_*0*_	206.45	22.96	< 0.001		
	So_LT_	*R*_*ref*_	2.44	0.06	< 0.001	0.24	1.19
		*E*_*0*_	166.41	13.99	< 0.001		
		R_*ref average*_	2.17	0.4			
		*E*_*0 average*_	179.8	90.89			

E_0_ is calculated using Eq (2) for each replicate of *Festuca* and *Sempervivum* plot measured in 2013, with standard error and P value. For each replicate, the modelling efficiency (MEF) and relative standard error (RSE) of the model are also given.

**Fig 5:**
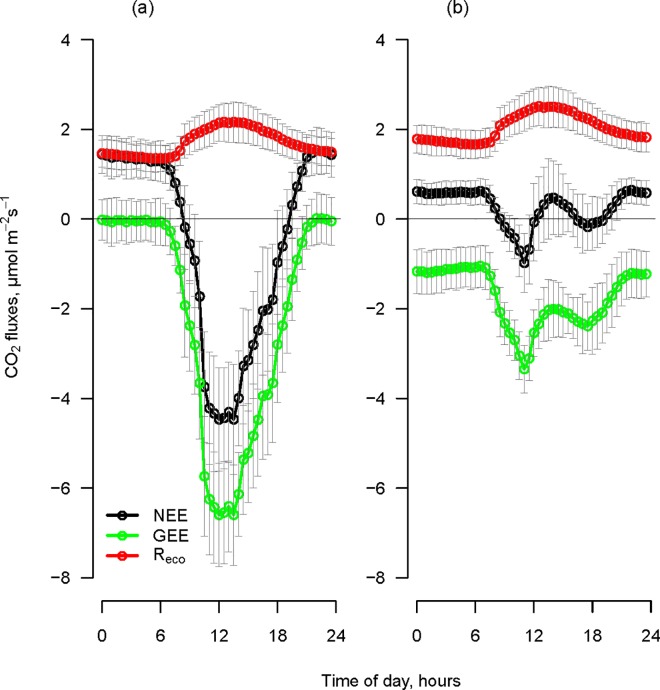
**Daily patterns (mean ± SD) during August 2013 of the net ecosystem exchange (NEE) measured at all the plots, the modelled ecosystem respiration (R**_**eco**_**) and gross ecosystem exchange (GEE) as obtained after flux partitioning for (a) *Festuca* and (b) *Sempervivum* plots.** GEE, indicating gross CO_2_ uptake, is represented here as negative consistently with the other fluxes.

The daily patterns of measured NEE and the values of R_eco_ and GEE obtained after flux partitioning in the month of August 2013 are shown in Fig 5. The maximum value of GEE (-6.6 ± 1.15 μmol CO_2_ m^-2^ s^-1^) for the *Festuca* plots in August was obtained at 12:00, the maximum value of GEE for *Sempervivum* plots (-3.35 ± 0.53 μmol CO_2_ m^-2^ s^-1^) was obtained around 11:00. Both vegetation types shared a very similar value of R_eco_, with the maximum value obtained around 12:30 (maximal R_eco_ for *Festuca* = 2.16 ± 0.43 μmol CO_2_ m^-2^ s^-1^; maximal R_eco_ for *Sempervivum* = 2.51 ± 0.43 μmol CO_2_ m^-2^ s^-1^).

### Seasonal pattern of NEE

**Fig 6:**
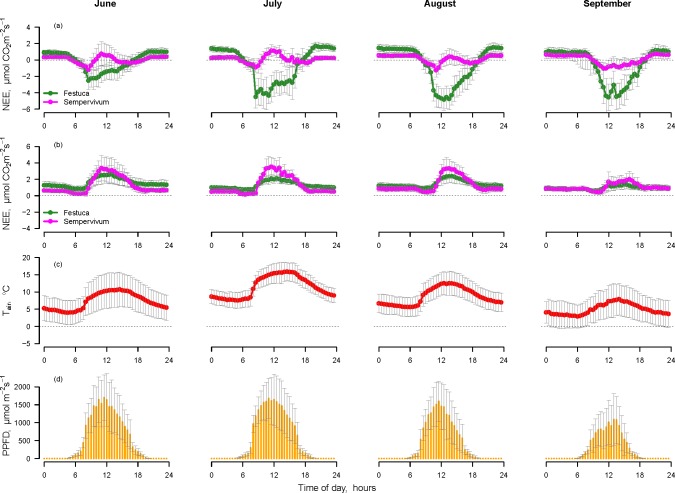
**Daily pattern of net ecosystem exchange (NEE) measured with (a) transparent chambers, (b) opaque chambers.** Panels (c) and (d) show the diurnal pattern of air temperature and photosynthetic active photon flux density (PPFD). Data are presented as monthly averages ± SD in June, July, August, and September 2013.

The data collected in the permanent plots over the 2013 vegetative period was used to analyse the NEE temporal trend of the two vegetation communities. Fig 6 shows, for each month, the daily pattern of NEE measured with transparent and opaque chambers, air temperature and PPFD. Data described in Fig 6 reflect a single measurement location for each community for transparent chambers (Fig 6A) and a different single measurement location for opaque chambers (Fig 6B). The daily trend measured in the *Festuca* plot with the transparent chamber showed a clear seasonal trend (Fig 6A). The intensity of assimilation fluxes increased during summer (maximal negative average value of NEE for the entire period recorded in August -4.47 ± 1.23 μmol CO_2_ m^-2^ s^-1^) and the assimilation peak underwent a shift from midmorning in June and July to noon in August and even later in September. The *Sempervivum* community showed a typical CAM daily pattern in June, July and August, with very similar NEE values during the same period of the day in the different months. The maximal negative average values of NEE measured during phase 2 was -1.18 ± 0.62 μmol CO_2_ m^-2^ s^-1^ in June; -0.69 ± 0.53 μmol CO_2_ m^-2^ s^-1^ in July and -0.98 ± 0.66 μmol CO_2_ m^-2^ s^-1^ in August. When the average temperature and PPFD decreased in September (Fig 6C and 6D), the morning peak of assimilation and the emission period around noon were no longer recognizable.

Measurements conducted with the opaque chambers (Fig 6B) showed that the two communities shared similar daily trends throughout the growing season, with a peak of respiration during the central hours of the day. The maximal positive average value of NEE for *Festuca* plots recorded in July was 2.66 ± 0.73 μmol CO_2_ m^-2^ s^-1^; the maximal positive average value of NEE for *Sempervivum* plots recorded in June was 3.04 ± 1.45 μmol CO_2_ m^-2^ s^-1^. The intensity of the fluxes in the two plant communities remained almost constant during the entire summer. *Festuca* plots always showed a higher intensity of emission during the night and *Sempervivum* plots showed a sharper peak during the day (Fig 6A and 6B). In September, the flux patterns of the two vegetation types largely overlapped.

### Seasonally cumulated NEE and NECB

**Fig 7:**
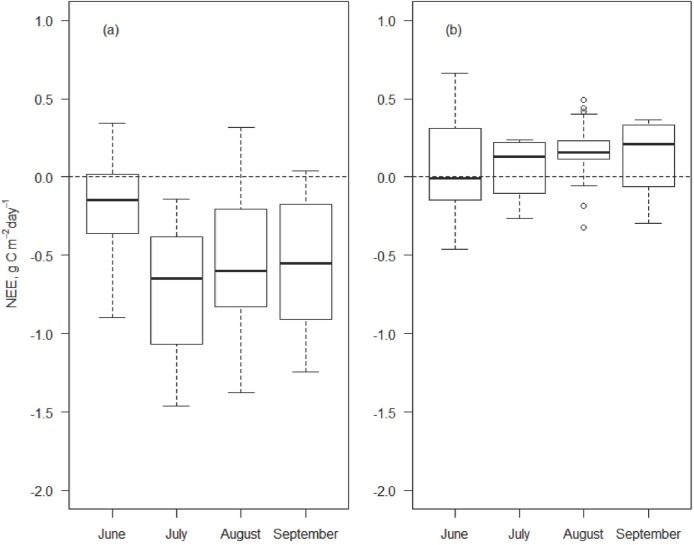
Box plots of cumulated daily values of net ecosystem exchange (NEE). Fluxes are measured with the long-term transparent chambers in 2013 (g C m^-2^ day^-1^) in (a) *Festuca* and (b) *Sempervivum* plots.

The cumulated value of NEE along the four months of study in 2013 (Fig 7), measured with transparent chambers in the permanent plots, was -45.8 and 11.9 g C m^-2^ for *Festuca* and *Sempervivum*, respectively. The cumulated values of GEE and R_eco_ resulting from NEE flux partitioning were -239.3 and 193.5 g C m^-2^ for the *Festuca* plot and -232.7 and 244.6 g C m^-2^ for the *Sempervivum* plot. By averaging the measured values of the permanent plots with the filled time series of the non-permanent plots of both plant communities, the cumulated value of NEE (mean ± SD) resulted -46.4 ± 35.5 and 31.9 ± 22.4 g C m^-2^, thus highlighting an opposite sink-source pattern of the two communities with respect to atmospheric CO_2_. GEE was -230.4 ± 50.3 and -186.5 ± 35.3 g C m^-2^ and R_eco_ was 184.0 ± 16.4 and 218.4 ± 38.9 g C m^-2^ for *Festuca* and *Sempervivum*, respectively.

The soil analysis of samples collected a few meters from the glacier tongue showed that total organic C in the upper 10 cm soil layer was < 150 g C m^-2^; therefore, we considered the total amount of C found in the soil-vegetation system in our plots on the LIA moraine as representative of the NECB of 160 years. Soil C in the upper 10 cm soil layer did not differ in plots dominated either by *Festuca* or by *Sempervivum* (Table 4). The total C content was measured as the average ± standard error of the C content in the soil and biomass of the five plots for each vegetation type, which was slightly higher in the *Sempervivum* than in the *Festuca* plots (2080 ± 230 and 1770 ± 130 g C m^-2^, respectively). The ANOVA test applied to the different categories into which the soil-plant system was divided showed statistically significant differences at the margins (p = 0.067) only for the quantity of C stored in the above-ground biomass (Table 4). Assuming that the amount of soil C at the beginning of the primary succession and soil C at a depth >10 cm were both negligible, the C in the upper soil and in the vegetation of *Sempervivum* and *Festuca* plots was accumulated through an average NECB of 13.0 and 11.1 g C m^-2^ y^-1^.

**Table 4:** Comparison of the C content in the plots of the two vegetation communities.

C content	*Festuca* (gCm^-2^)	*Sempervivum* (gCm^-2^)	*P value*
Soil	1073.87 ± 117.22	1360.2 ± 205.57	0.260
Above-ground biomass	195.98 ± 18.19	282.70 ± 35.62	0.062
Necromass	66.58 ± 5.81	62.59 ± 11.19	0.760
Below-ground biomass	431.71 ± 63.05	375.37 ± 25.86	0.430
Total soil plant system	1768.15 ± 128.58	2080.86 ± 234.75	0.276

Soil (0–0.1 m) roots and aboveground vegetation inside the plots are considered (total n of samples = 40). Values are reported as averages of five samples ± standard error for each category in which the soil-plant system was divided. P values given are the result of the ANOVA test with 8 Degrees of Freedom after the normality of distribution and homogeneity of the data variance were ascertained.

## Discussion

The present study is a first contribution to the C budget calculation of glacier forefields. In particular, we want to understand if the extremely patchy characteristic of the vegetation plays a role in the calculation of the total C budget of the deglaciated ecosystem. The observed CO_2_ fluxes that determine final C budget are the result of the overall interactions between the different plant communities and their environment. At the one hand, several morphological and physiological plant traits, including the photosynthetic pathway, are known to play a role in capturing and converting the solar radiation [33,54]; at the other hand, several chemical, physical and microbiological features are also involved in the respiration and in the long term carbon release processes [55]. To understand if different vegetation communities are characterized by different CO_2_ fluxes, the present study analysed separately the CO_2_ fluxes of two vegetation communities growing on the same alpine moraine. The selected study plots were characterized by vegetation showing a different C assimilation pathway, i.e. a *Festuca* community dominated by C3 species and a *Sempervivum* one dominated by a weak CAM species. Robust differences in CO_2_ fluxes between the two vegetation types, both in daily and seasonal patterns, were detected by capturing NEE measurements every half hour over the vegetation season. Through the partitioning of NEE we derived the CO_2_ assimilation and release processes of the two vegetation communities, at scales from the half hour to the season, and by the analysis of C accumulation, we evaluated the long term NECB.

Measurement conducted with opaque and transparent chambers showed the daily pattern of NEE for the two vegetation communities. When the NEE was measured with clear chambers, *Festuca* plots showed a daily pattern similar to that registered for other alpine grasslands [15,56], although with a slightly lower intensity of fluxes. The daily pattern of *Sempervivum* plots measured with the transparent chambers in the field reproduced the phases described and tested by Osmond [48] under controlled conditions, but with interesting differences. In general, *Sempervivum* plots performed like weak CAMs, as described by Silvera et al. (2014) for tropical orchids, with a greater CO_2_ uptake occurring in the light period than in the dark period. The weak CAM behaviour was confirmed by observing the C isotopic ratio found in the biomass of *Sempervivum* plots. The observed isotopic ratio in the vegetation (δ^13^C = -21.18 ± 0.41) represents the typical value that weak CAM plants display in the condition of middle range temperature and under no particular water stress [21,37].

Sempervivum plots showed a daily NEE pattern characterized by a first maximum of assimilation during the morning and a second maximum of assimilation in the evening. During the night, C uptake was limited, which is uncommon in CAMs. This pattern can be largely explained by the intrinsic characteristics of PEPC, the enzyme regulating the CAM photosynthetic pathway and many other environmentally related plant functions, like seed germination, carbon accumulation in fruits, etc. [57]. Recent biochemical studies found that PEPCs belong to a multigene family encoding several plant-type PEPCs. PEPC activity can be regulated according to a circadian clock and modulated according to environmental stresses [58,59]. The regulatory gene expression can be influenced by several environmental parameters, including the level of salinity, the light intensity at the different wave bands [60], and the temperature. As a results of the specific features of PEPCs, CAM plants are very plastic organisms, as they finely regulate the amplitude of malic acid change and the amount of day versus night CO_2_ fixation in relation to the peculiar combination of internal conditions and environmental factors [46]. Therefore, the daily pattern of NEE in *Sempervivum* plots we presented here is peculiar, and compared to the chart proposed by Osmond [48], shows an upper shift to a higher emission rate of CO_2_. During phase 1, when CAM plants are supposed to show nocturnal carbon assimilation [33], we recorded some net CO_2_ emissions. As described by Wagner and Larcher [37], *S*. *montanum* shows a clear nightly CO_2_ uptake when large differences in air temperature between day and night occur. In our study area, the diurnal air temperature fluctuation never exceeded 15°C, although the real temperature measured in the rosettes of the plant may have reached higher values [21]. However, considering the measurements conducted with the opaque chambers, we observed that night values of NEE of the *Sempervivum* plots were always lower than those of the *Festuca* plots, presumably because nocturnal carbon assimilation was also occurring. Past studies conducted in the laboratory under controlled conditions suggest that it is almost impossible to find a temperature threshold that allows the switch from the emission to the assimilation process in the CAM cycle. The regulation of the CAM cycle does not directly depend on the current environmental conditions, but on the internal reserve of malic acid generated in previous days [37].

The two vegetation communities exhibited contrasting seasonal patterns. *Festuca* plots showed a more marked seasonality than did *Sempervivum*, with the highest sink observed in August. Instead, the daily course of *Sempervivum* was almost constant from June to August, thus displaying its typical weak CAM behaviour. Interestingly, in September, when the average temperature is usually around 10°C, the CAM metabolism was no longer evident and the midday reduction in NEE was no longer appreciable. Presumably, *S*. *montanum* behaved as a C3 plant. As a result, the daily pattern of *Sempervivum* plots, measured with opaque and transparent chambers, became similar to those of *Festuca* plots, although with lower maximal net uptake.

To partition NEE fluxes into the originating R_eco_ and GEE fluxes, night time NEE data are generally used to extrapolate R_eco_ during the day by adopting a temperature response function [50], although it has been suggested that this method may lead to an overestimation of R_eco_ [61]. Alternatively, the light response curve estimated during daytime is used, and the respiration is obtained from the intercept on the ordinate [62]. In the case of vegetation communities showing CAM metabolism, CO_2_ assimilation flux and photosynthesis are temporally separated, and we could not apply the customary methodologies to *Sempervivum* plots. In fact, NEE night data describe only part of the assimilation process, and daytime NEE data are weakly coupled with illumination. The combined use of transparent and opaque chambers allowed us to detect the Osmond phase 3 occurring during the central part of the day. Data corresponding to this phase were used to establish a temperature response function and to model R_eco_ during the whole day. With this flux partitioning method, we were able to model the total CO_2_ uptake in *Sempervivum* plots, but we could not define the trend in time of re-fixation of internally released CO_2_ derived from malate degradation. In C3 species we first compared the temperature response function obtained by using the customary methodology (night data) and data of phase 3, and we found no statistical difference. Therefore, for methodological coherence, we preferred to use the same day period, phase 3, (equal to the same temperature interval) to find the temperature response function and to model R_eco_ also for the C3 species. To confirm the findings obtained through this flux partitioning methodology, a straightforward method would be the isotopic discrimination of the respired CO_2_, possibly with the Bayesian modelling approach as described by Ogle and Pendall [63].

The results of flux partitioning gave reproducible values for both vegetation communities and for all the trial periods. The two vegetation types, exhibiting different assimilation mechanisms, also showed different levels of daily maximal assimilation. Despite this fact, the vegetation plots considered were characterized by similar emission patterns. An analogous result was found by Wohlfahrt et al. [27], who considered the influence of species physiology by modelling the C budget of three meadows differing in land management. Similar results have also been shown in comparing different tundra vegetation communities, where variations in NEE are more strongly driven by differences in assimilation than in respiration rates [30]. The NEE observed during the growing season indicates that the vegetation characterized by *F*. *halleri* acted as a sink of C in the emergent ecosystem, while the scattered plant community mainly characterized by the presence of *S*. *montanum* was a source of CO_2_ during the measured period. A similar CO_2_ source was also found for arctic vegetation in harsh habitats [16,64]. Relatively dry tundra, which is similar to our ecosystem with regard to climatic conditions, was proved to act mainly as a C source [65]. Peatland ecosystems in mountain environments are also subject to a yearly switch from source to sink of C depending on soil moisture [24,66]. Alpine grasslands can also act as both a sink and a source owing to changes in land management [67]. Inter-annual variability in the C balance, very typical of grassland communities with low productivity, is mainly linked to yearly fluctuations of soil respiration depending on temperature and water availability [68]. In fact, below-ground respiration of plant communities in glacier forefields can significantly contribute to soil CO_2_ efflux due to high allocation rates of C in the below-ground organs [8,69].

The amount of C that has accumulated in both ecosystem types after 160 years is in the same range as those found for other glacier forefields [70,71]. Since measurements performed during the growing season highlighted a much larger assimilation in the C3 than in the CAM dominated community, we could have expected a similar difference in the NECB, which was found conversely to be not statistically different. To understand the discrepancy between the short-term NEE and the long term NECB we should consider the processes differentially affecting the fate of the recently accumulated carbon in the two ecosystems. For example, direct removal of herbs, and not of rosettes, from selective herbivorous feeding can explain a significant portion of this discrepancy. Moreover, we should consider that while grasslands are easily prone to DIC and DOC leaching [12], rosettes undergo to a larger internal recycling during the decomposition of old organic material [21] which minimizes the amount of C with low ^13^C enrichment released into the soil. These two processes are part of the non-gaseous lateral fluxes differentiating NECB from NEE, as described by Chapin et al. [9].

Moreover, we should take into account the representativeness of the considered vegetation communities and their measured fluxes in a broader temporal time range. In fact, we measured NEE only during the vegetative season, while winter C losses may be substantial [72]. The composition and quantity of the organic matter originated by the two communities and their associated compounds, such as lignin and secondary metabolites, possibly influences the rate of decomposition and soil winter respiration [62,63]. It has been shown that high gross primary productivity values are not always coupled with high rates of C accumulation in the ecosystem. Annual grasses such as *F*. *halleri* provide a consistent quantity of fresh material as the substrate for heterotrophic respiration that diminishes the C retention capacity of the ecosystem [73]. By analysing a larger, multiannual scale, we should consider that both *Festuca* and *Sempervivum* plots belong to a late stage of the ecological process of primary succession, and, in this study, we didn’t gain indications on the biogeochemical processes occurred at the same locations during the earlier stages of the vegetation establishment. The C isotopic ratio observed in the soil, which was almost the same for the two communities in spite of the different assimilation pathways, is consistent with these interpretations.

In conclusion, this study reports on the comparative analysis of the CO_2_ fluxes of two alpine plant communities living on a glacier forefield presenting different pathways of assimilation, a typical C3 grassland and a succulent CAM rosettes community. We demonstrate the feasibility of the combined use of opaque and transparent chambers to assess the NEE, R_eco_ and GEE also for CAM plants. We show that data collected with opaque chambers during the Osmond phase 3, the central part of the day, can be used to model the relation of R_eco_ with temperature.

The comparative analysis demonstrates that the C dynamics of the two vegetation communities are quite distinct. The photosynthetic pathway of the dominant species is correlated with large differences in the C cycle. The two plant communities investigated showed similar respiratory patterns (R_eco_) but had different assimilation patterns (GEE) because of their response to the climatic constraints, in particular to light intensity. The different communities showed contrasting NEE, which was found to be positive for CAM rosettes and negative for C3 plants. We found that the establishment of vegetation types having different physiology can be regarded as an eigenvalue in the carbon accumulation dynamics at time scales shorter than one year.

The overall NECB obtained from soil and vegetation analyses was similar in the two vegetation types. The respiration and lateral fluxes acted as negative feedback to the increase of sequestered carbon, and differently from the case of tree and herbaceous ecotones, tend to minimize the differences in the long-term NECB.

The inconsistency between seasonal and interannual balance of the two vegetation communities indicates that for an accurate C budget of the glacier forefield ecosystem, moving from an established GEE value, it is important to monitor the microbial processes dominating during the dormant season and the lateral flows during the different stages of the primary succession. The analysis of the C fluxes of different vegetation communities is interesting to understand the processes involved in the evolution of the ecosystem, but not in the mere assessment of NECB, since in the long term respiration and disturbances tend to equilibrate the different observed GEE.

## Supporting Information

S1 Fig Example of the experimental setup in 2012. Only the set-up used during the first three measurement periods is shown. Each measurement period lasted three days.
(DOCX)

S2 Fig Example of the experimental set-up in 2013. Only the set-up used during the first three measurements periods is shown. Each measurement period lasted one week.
(DOCX)

S3 Fig Example of raw CO2 data (1 Hz) collected during one measurement with a clear chamber over a *Festuca* plot. X axis represents time after chamber closure (s). Fluxes were recomputed considering only the data measured between 20 and 60 seconds after chamber closure (limits represented in the plot by the green and red vertical lines). In such a short time the exponential fitting produced an uncommon high value of CO2 uptake (-9.33 μmol CO_2_ m s^-1^) compared with the linear fitting (-2.90 μmol CO2 m^2^s^-1^) or compared with the exponential fitting if the regression time was enlarged to a 20–100 s time window (- 2.63 μmol CO_2_ m^2^s^-1^).
(DOCX)
