# Supplementary material for: Colonization of a Deglaciated Moraine: Contrasting Patterns of Carbon Uptake and Release from C3 and CAM Plants
Source: PLoS One. 2016 Dec 29;11(12):e0168741. doi: 10.1371/journal.pone.0168741 (PMC5199236; doi:10.1371/journal.pone.0168741)
Supplement: S2 Fig — Only the set-up used during the first three measurements periods is shown. Each measurement period lasted one week. (DOCX) [file pone.0168741.s002.docx]

**Colonization of a deglaciated moraine: contrasting patterns of carbon uptake and release from C3 and CAM plants**

Elisa Varolo, Damiano Zanotelli, Leonardo Montagnani, Massimo Tagliavini, Stefan Zerbe

**Supplemental material S2**


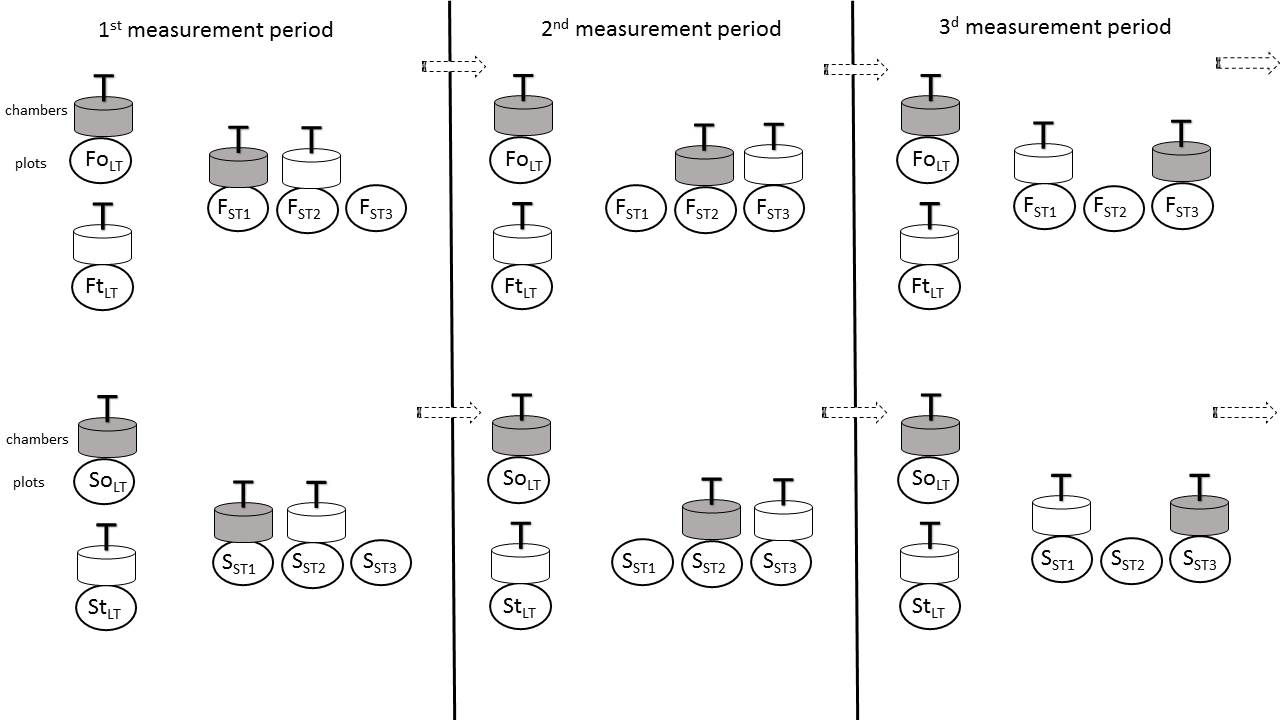


**S2 Fig**. **Example of the experimental set-up in 2013**. Only the set-up used during the first three measurements periods is shown. Each measurement period lasted one week.
