# Supplementary material for: Colonization of a Deglaciated Moraine: Contrasting Patterns of Carbon Uptake and Release from C3 and CAM Plants
Source: PLoS One. 2016 Dec 29;11(12):e0168741. doi: 10.1371/journal.pone.0168741 (PMC5199236; doi:10.1371/journal.pone.0168741)
Supplement: S3 Fig — X axis represents time after chamber closure (s). Fluxes were recomputed considering only the data measured between 20 and 60 seconds after chamber closure (limits represented in the plot by the green and red vertical lines). In such a short time the exponential fitting produced an uncommon high value of CO2 uptake (-9.33 μmol CO2 m s-1) compared with the linear fitting (-2.90 μmol CO2 m2s-1) or compared with the exponential fitting if the regression time was enlarged to a 20–100 s time window (- 2.63 μmol CO2 m2s-1). (DOCX) [file pone.0168741.s003.docx]

**Colonization of a deglaciated moraine: contrasting patterns of carbon uptake and release from C3 and CAM plants**

Elisa Varolo, Damiano Zanotelli, Leonardo Montagnani, Massimo Tagliavini, Stefan Zerbe

**Supplemental material S3**


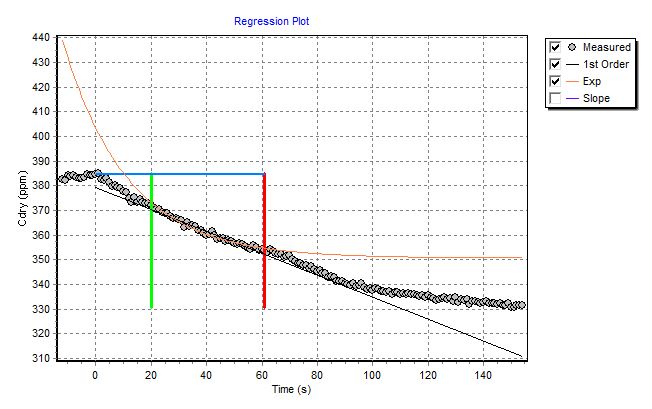


**S3 Fig. Example of raw CO_2_ data (1 Hz) collected during one measurement with a clear chamber over a *Festuca* plot**. X axis represents time after chamber closure (s). Fluxes were recomputed considering only the data measured between 20 and 60 seconds after chamber closure (limits represented in the plot by the green and red vertical lines). In such a short time the exponential fitting produced an uncommon high value of CO_2_ uptake (-9.33 µmol CO_2_ m^2^s^-1^) compared with the linear fitting (-2.90 µmol CO_2_ m^2^s^-1^) or compared with the exponential fitting if the regression time was enlarged to a 20-100 s time window (- 2.63 µmol CO_2_ m^2^s^-1^).
